# Application of spin-crossover water soluble nanoparticles for use as MRI contrast agents

**DOI:** 10.1038/s41598-018-33362-6

**Published:** 2018-10-08

**Authors:** Asami Tsukiashi, Kil Sik Min, Hikaru Kitayama, Hiroaki Terasawa, Sosuke Yoshinaga, Mitsuhiro Takeda, Leonard F. Lindoy, Shinya Hayami

**Affiliations:** 1 Department of Chemistry, Graduate School of Science and Technology, Kumamoto University, 2-39-1 Kurokami, Chuo-ku, kumamoto 860-8555 Japan; 2 Department of Chemistry Education and Green-Nano Materials Research Center, Kyungpook National University, Daegu, 41566 Republic of Korea; 3 Department of Structural BioImaging, Faculty of Life Sciences, Kumamoto University, 5-1 Oe-honmachi, Chuo-ku, Kumamoto 862-0973 Japan; 4 School of Chemistry, The University of Sydney, Sydney, NSW 2006 Australia; 5 Institute of Pulsed Power Science (IPPS), Kumamoto University, 2-39-1 Kurokami, Chuo-ku, Kumamoto 860-8555 Japan

## Abstract

Water soluble spin-crossover (SCO) iron(II) nanoparticles (NPs) were synthesized by the polyethylene glycol (PEG) coating of [Fe(Htrz)_3-3×_(NH_2_trz)_3×_](BF_4_)_2_ (x = 0, 0.1, 0.5 and 1). The NPs with x = 0.1 show gradual SCO behavior over 280–330 K in water. The relaxation times, *T*1 and *T*2, were determined and the thermally-responsive *T*2 values making these NPs a candidate for use as a MRI contrast agent.

## Introduction

Magnetic resonance imaging (MRI) is a useful diagnostic medical tool for the non-invasive visualization of internal body parts. For the imaging, the hydrogen nucleus is used because of its abundance in water and fat. The time taken for the protons to fully relax is measured in two ways. The first is the time taken for the magnetic vector to return to its resting state and the second is the time needed for the axial spin to return to its resting state. The first is called T1 relaxation, the second is called T2 relaxation. A MRI contrast agent is normally employed to enhance the MRI image. Commonly, Gd-chelate compounds^1^ and iron oxide materials^2,3^ are used as MRI contrast agents. The Gd-chelate compounds are especially effective owing to the large spin quantum number of Gd(III) (*S* = 7/2). Nevertheless, nephrogenic systemic fibrosis (NSF) is known as a serious kidney disease that can result from the use of such Gd-chelate contrast agents^4–6^. This provides a motivation for the development of new contrast agents not based on Gd(III).

**Figure 1:**
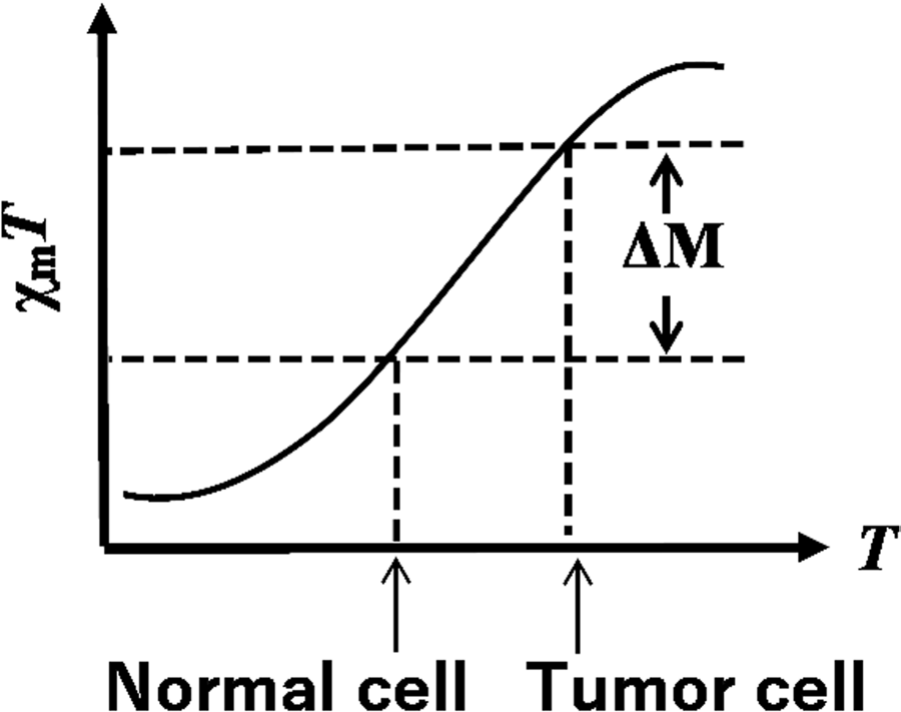
Application of SCO to MRI.

Recent studies have demonstrated that SCO materials can potentially be employed as MRI contrast agents^7,8^. Spin crossover (SCO) materials undergo a change in their spin state between high-spin (HS) and low-spin (LS) on exposure to external stimuli, i.e., temperature, pressure, magnetic field, and light irradiation. Such materials have been intensively investigated for use in applications such as information storage, sensor development, and display technologies^9–11^. SCO iron(II) complexes undergo a spin state change (ΔM in Fig. 1) between LS (*S* = 0) and HS (*S* = 2) that is temperature dependent. Generally, tumor cells are more thermally-sensitive than normal cells, and the temperature of tumor cells can be readily increased by means of hyperthermia treatment^12,13^. In view of this, SCO materials can in principle be employed as MRI contrast agents by using the change of their magnetism with temperature when the temperature of tumor cells is higher than nomal cells.

In this work, we focused on the use of iron(II) SCO materials of type [Fe(Htrz)_3-3×_(NH_2_trz)_3×_](BF_4_)_2_ (Htrz = 1,2,4-1H-triazole, NH_2_trz = 4-NH_2_-1,2,4-triazole, and x = 0, 0.1, 0.5 and 1) as candidates for use as new contrast agents^14–16^. For practical application in MRI, water dispersion is important and to this end we synthesized the SCO nanoparticles (NPs) with a PEG coating. Here, we report a study of these thermally-responsive MRI contrast agents based on the SCO NPs.

Magnetic susceptibility measurements for the NPs with x = 0, 0.1, 0.5 and 1 in aqueous dispersion were performed by NMR measurements. The *χ*_m_*T* values (*χ*_m_, molar magnetic susceptibility; *T*, temperature) were estimated by the Evans method employing eq. (1) below (Fig. S5)^17–19^; the calculation parameters are given in Table S2. All NPs showed gradual SCO behaviour over the temperature range 283–323 K.

1 $${\chi }_{M}^{P}=\frac{3{\rm{\Delta }}f{M}^{P}}{4\pi fm}+{\chi }^{0}{M}^{P}+\frac{{\chi }^{0}{M}^{P}({d}_{0}-{d}_{s})}{m}-{\chi }_{M}^{dia}$$

${\chi }_{M}^{P}$: Molar paramagnetic susceptibility in cm^3^ mol^−1^.

∆*f*: Frequency difference between the two peaks of inner and outer tube in Hz.

*M*^*P*^: Molecular weight of the substance in g mol^−1^.

*f*: Frequency of NMR instruments in Hz.

*m*: Mass of the substance in 1 mL of solution in g mL^−1^.

${\chi }^{0}$: Mass susceptibility of the solvent in cm^3^ mol^−1^.

${d}_{0}$: Density of the solvent in g cm^−3^.

${d}_{s}$: Density of the solution in g cm^−3^.

${\chi }_{M}^{dia}$: Diamagnetic correction for the magnetic susceptibility in cm^3^ mol^−1^.

## Results

**Figure 2:**
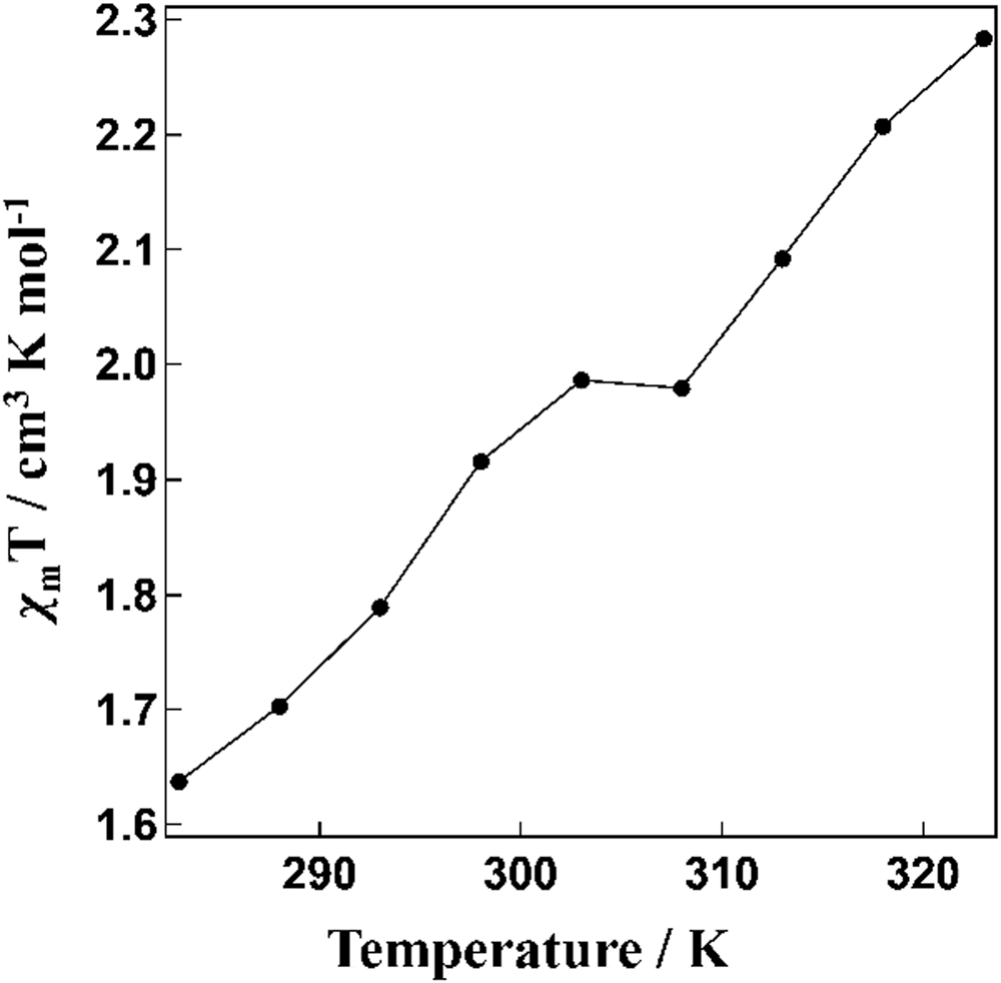
*χ*_m_*T* vs *T* plot for NPs **1** in water.

**Figure 3:**
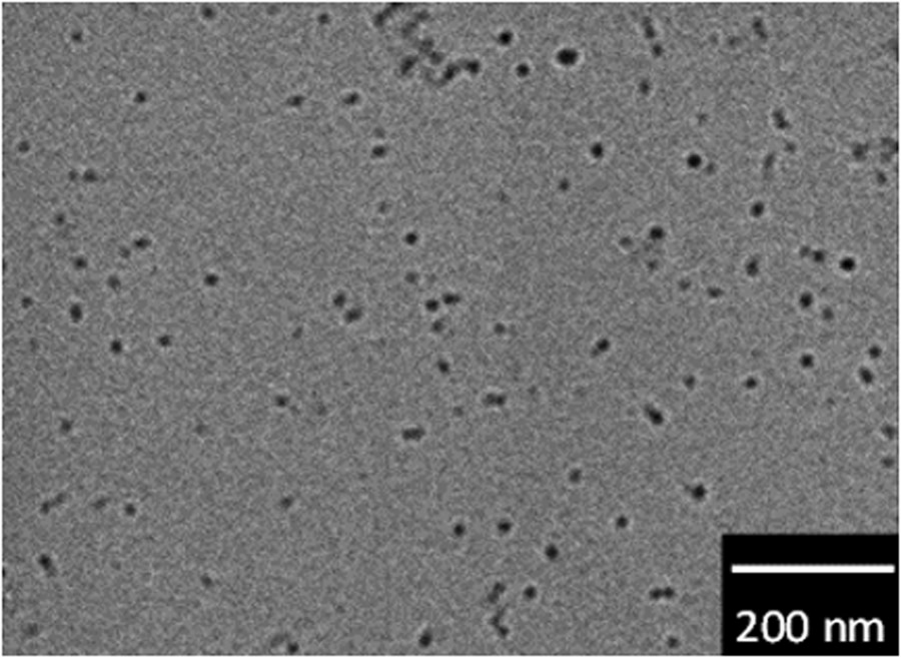
TEM image of NPs **1**.

**Figure 4:**
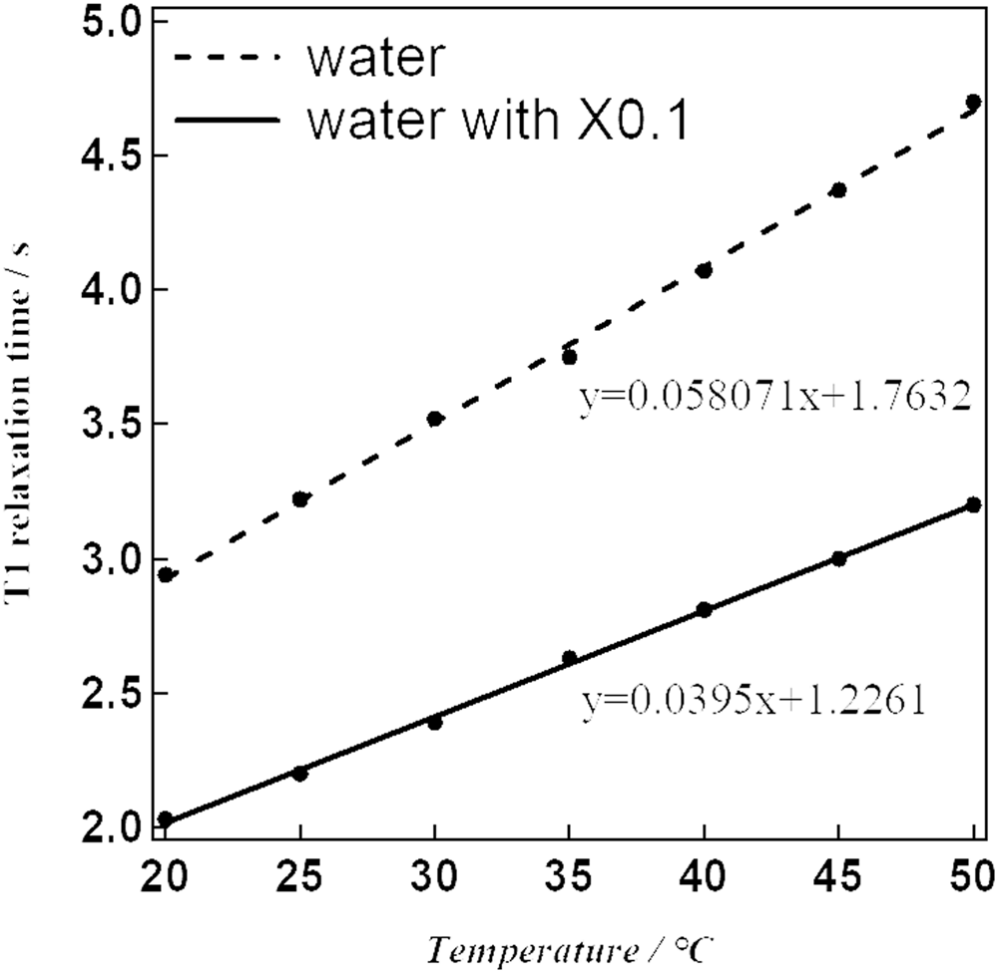
*T*1 relaxation time vs temperature plot.

**Figure 5:**
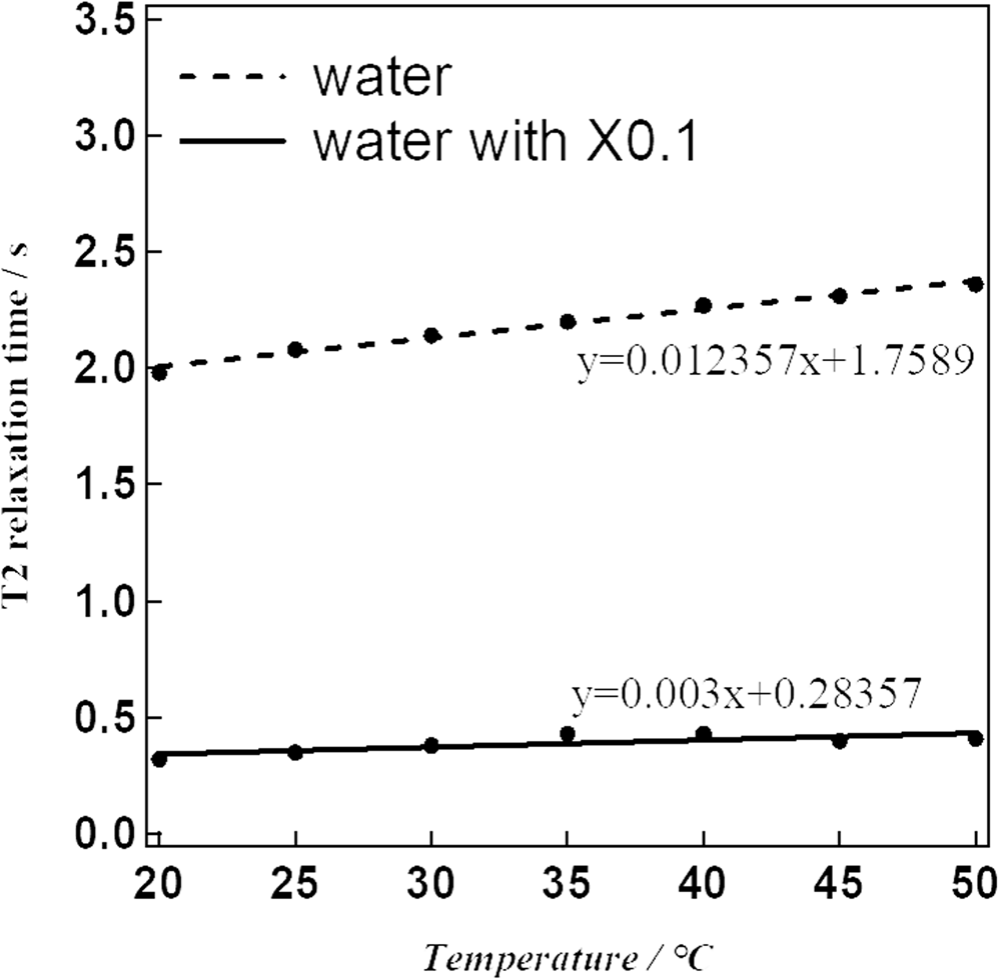
*T*2 relaxation time vs temperature plot.

Of all the NPs investigated, [Fe(Htrz)_2.7_(NH_2_trz)_0.3_](BF_4_)_2_ (**1**) exhibited the steepest SCO behaviour (Fig. 2; that is, it showed the largest temperature dependence of *χ*_m_*T*). Hence, we employed this NP derivative for use in our MRI investigation. The synthesized NPs **1** were dispersed in water and measured by transmission electron microscopy (TEM) to confirm that nanoparticulation had occurred. As estimated by TEM, the particle size was mainly around 20 nm (Fig. 3). The powder X-ray diffraction (PXRD) patterns for compound **1** and its corresponding NPs (NPs **1**) were determined at room temperature. The patterns for **1** and its NPs are almost the same, in accord with the NPs being successfully synthesized with the structure of **1** being retained intact (Fig. S2). NPs **1** were dispersed in water and investigated by NMR to obtain the *T*1 and *T*2 relaxation times (Figs 4 and 5). In the absence of NPs, water molecules gave a *T*1_0_ relaxation of 2.94 s at 20 °C. On the other hand, the *T*1 relaxation time was 2.03 s for the aqueous dispersion of NPs at 20 °C. NMR measurements were also carried out at higher temperatures: 50 °C for water and the aqueous dispersion of NPs. The *T*1_0_ relaxation time for water was 4.70 s (50 °C), while for the aqueous dispersion of NPs it was *T*1 = 3.20 s (50 °C). The correlation between *T*1_0_ and *T*1 relaxation times vs. temperature is shown in Fig. 4. We observed that the *T*1 relaxation time of water molecules with or without any contrast agent becomes longer with increasing temperature. The slope of the linear *T*1 vs *T* plot for water is 0.1215 (Fig. 4). However, in the case of the aqueous dispersion of NPs, the slope is only 0.0395. This difference between the slopes indicates that the presence of the NPs strongly shortens the *T*1 relaxation time at higher temperature (50 °C) compared to the lower temperature (20 °C). As such, these results demonstrate that a temperature-dependent shortening effect on the proton relaxation time occurs. The relaxivity (*r*) of the MRI contrast agent was calculated using equations (2 and 3).

2 $$\frac{1}{T{1}_{0}}=\frac{1}{T1}-{r}_{1}\,[{\rm{C}}]$$

3 $$\frac{1}{T{2}_{0}}=\frac{1}{T2}-{r}_{2}\,[{\rm{C}}]$$

*T*1_0_: *T*1 relaxation time for original sample (s).

*T*1: *T*1 relaxation time shortened by contrast agent (s).

*r*_1_: Relaxivity for *T*1 relaxation time (mM^−1^ s^−1^).

*T*2_0_: *T*2 relaxation time for original sample (s).

*T*2: *T*2 relaxation time shortened by contrast agent (s).

*r*_2_: Relaxivity for *T*2 relaxation time (mM^−1^ s^−1^).

C: Concentration of contrast agent (mM).

## Discussion

The *T*1 relaxivity (*r*_1_) values were 0.16 mM^−1^ s^−1^ at 20 °C and 0.10 mM^−1^ s^−1^ at 50 °C. The corresponding proton relaxation time of water at 50 °C was calculated using the linear equation given in Fig. 4; the calculation parameters are listed in Table 1. The calculated *r*_1_ value is far from that for the commonly used MRI contrast agent, Gd-DTPA^20^. *T*2 relaxation times were also obtained by NMR measurements (Fig. 5). At 20 °C, the values were 1.98 for water and 0.32 s for the aqueous dispersion of NPs. In higher temperature experiments, the *T*2 relaxation times were 2.36 s for water at 50 °C and 0.41 s for the aqueous dispersion of NPs at 50 °C. The slope of the linear plot for the *T*2 relaxation time of water was 0.003. While for the aqueous dispersion of NPs, the slope was effectively zero. As for the *T*1 relaxation times, this result shows that the presence of the NPs strongly shortens the *T*2 proton relaxation time at the higher temperature (50 °C). The calculated *T*2 relaxivities (*r*_2_) were 2.44 mM^−1^ s^−1^ at 20 °C and 1.91 mM^−1^ s^−1^ at 50 °C, respectively. The calculated parameters are shown in Table 1. The ratio of transverse/longitudinal relaxivity, *r*_2_/*r*_1_, was found to be 19.1 at 50 °C, which seems an effective value for the use as a negative contrast agent.

**Table 1:** Calculation parameters for *T*1 relaxivity (*r*_1_) and *T*2 relaxivity (*r*_2_). Concentration; 1.0 × 10^−3^ M.

Temperature	*T*1_0_	*T*1	*r*_1_	*T*2_0_	*T*2	*r*_2_
20 °C	2.94	2.03	0.16	1.98	0.32	2.44
50 °C	4.7	3.2	0.10	2.36	0.41	1.91

In conclusion, we have synthesized the iron(II) triazole NPs (**1**). The NPs showed gradual spin transition around 300 K in aqueous dispersion and temperature-dependent shortened proton relaxation times. The calculated *r*_2_/*r*_1_ value indicates that NPs is a candidate for use as a thermally-responsive *T*2 *s*hortening MRI contrast agent.

## Methods

### Physical measurements

The magnetic susceptibility in the solution was carried out with a JEOL (500-ECX) instrument operating at 500 MHz by the Evens method using D_2_O. The inner tube was filled with 1% *t*-BuOH in D_2_O solution. The NPs were dispersed in D_2_O solution containing 2% *t*-BuOH, and the dispersion liquid was added to the outer tube. Transmission electron microscopy (TEM) was performed to determine particle sizes (JEOL, 2000FX, 200 kV). NPs were dispersed in water and deposited on a holey carbon film. Powder X-ray diffract grams were recorded on a Rigaku X-ray diffractometer (RAD-2A with a 2.0 kW CuKα X-ray). The *T*1 and *T*2 relaxation times were performed on ^1^H NMR spectroscopy. The experiments were carried out with a Bruker AvanceIII at 500 MHz. The water sample was measured with the aid of double-walled NMR tube. The water sample was prepared by H_2_O was put in the inner tube and 99.8% of D_2_O was added to the outer tube. The NPs were dissolved in mixed solution 97% H_2_O and 3% D_2_O at a concentration of 1 mM and measured with single NMR tube. The magnetic susceptibilities in the solid state for NPs and bulk samples were measured between 100 K and 400 K with a superconducting quantum interference device (SQUID) magnetometer (Quantum Design MPMS-XL). Atomic absorption analysis was performed to measure the ratio of SCO complex in the NPs with an atomic absorption spectrometer (PerkinElmer, PinAAcle 500). Each NP sample (10 mg) was dissolved in 100 mL of 0.1 M nitric acid aqueous solution before measurement (Fig. S1 and Table S1).

## Electronic supplementary material

SUPPLEMENTARY INFORMATION
