# Supplementary material for: Application of spin-crossover water soluble nanoparticles for use as MRI contrast agents
Source: Sci Rep. 2018 Oct 8;8:14911. doi: 10.1038/s41598-018-33362-6 (PMC6175957; doi:10.1038/s41598-018-33362-6)
Supplement: Supplementary file 1 — SUPPLEMENTARY INFORMATION [file 41598_2018_33362_MOESM1_ESM.docx]

**Supporting Information**

Application of spin-crossover water soluble nanoparticles for use as MRI contrast agents

Asami Tsukiashi,^1^ Kil Sik Min,^2^ Hikaru Kitayama,^1^ Hiroaki Terasawa,^3^ Sosuke Yoshinaga,^3^ Mitsuhiro Takeda,^3^ Leonard F. Lindoy^4^ and Shinya Hayami*^,1,5^

^1^ Department of Chemistry, Graduate School of Science and Technology, Kumamoto University, 2-39-1 Kurokami, Chuo-ku, kumamoto 860-8555 Japan.

^2^ Department of chemistry Education and Green-Nano Materials Research center, Kyungpook National University, Daegu 41566, Republic of Korea.

^3^ Department of Structural BioImaging, Faculty of Life Sciences, Kumamoto University, 5-1 Oe-honmachi, Chuo-ku, Kumamoto 862-0973, Japan.

*^4^ School of Chemistry, The University of Sydney, NSW 2006, Australia*

*^5^Institute of Pulsed Power Science (IPPS), Kumamoto University, 2-39-1 Kurokami, Chuo-ku, Kumamoto 860-8555, Japan.*

E-mail: hayami@kumamoto-u.ac.jp

**Figure S1**. Calibration curve of iron standard solution (dot; measured value, dotted line; approximate straight line).

**Table S1**. AAS analysis of **1**. Fe content in 10mg of NPs

| Sample | Fe content (ppm) | Complex concentration (%) |
| --- | --- | --- |
| X0.1 | 8.73 | 55.4 |

**Table S2**. peak shift, molar paramagnetic susceptibility and magnetic susceptibility of **1** by Evans method.

| K | ∆f | $\chi_{M}^{P}$ | *χ*_m_T |
| --- | --- | --- | --- |
| 283 | 0.045 | 0.005785 | 1.637074 |
| 288 | 0.046 | 0.005913 | 1.703020 |
| 293 | 0.048 | 0.006106 | 1.789084 |
| 298 | 0.050 | 0.006427 | 1.915383 |
| 303 | 0.051 | 0.006556 | 1.986471 |
| 308 | 0.050 | 0.006427 | 1.979658 |
| 313 | 0.052 | 0.006685 | 2.092267 |
| 318 | 0.054 | 0.006942 | 2.207447 |
| 323 | 0.055 | 0.007070 | 2.283677 |

**Figure S2.** PXRD pattern of bulk and NPs

**Figure S3**. *χ*_m_*T* vs *T* plot for NPs x=0 in water solution.

**Figure S4**. *χ*_m_*T* vs *T* plot for NPs x=0.5 in water solution.

**Figure S5**. *χ*_m_*T* vs *T* plot for NPs x=1 in water solution.

**Figure S6**. Magnetic susceptibility for complex **1**.

(▲) : Bulk heating, (▼) : Bulk cooling, (▲) : NPs heating, (▼) : NPs cooling
